# Analysis of the potential profile and influencing factors of nurses’ change fatigue in “Internet+Nursing Services”

**DOI:** 10.3389/fpubh.2026.1758698

**Published:** 2026-03-24

**Authors:** Yaqin Hu, Mengying Wang, Jinghua Yi, Jing Li

**Affiliations:** 1 International School of Clinical Medicine, Lishui University, Lishui, China; 2 Medicine School of Lishui University, Lishui, China; 3 International School of Clinical Medicine, Lishui University (Lishui Central Hospital), Lishui, China

**Keywords:** “Internet+Nursing Services”, change fatigue, influencing factors, nurses, potential profile features

## Abstract

**Purpose:**

To explore the potential profile of change fatigue in “Internet+Nursing Services” and analyze its influencing factors.

**Participants and methods:**

We recruited 650 “Internet+Nursing Services” nurses from 5 hospitals in Li Shui City between March and April 2025 using convenience sampling. Data were collected using a general information questionnaire, a change fatigue scale, and the “Internet+Nursing Service” professional identity scale.

**Results:**

The change fatigue of nurses in “Internet+Nursing Services” was divided into three groups: low change fatigue (40.3%), moderate change fatigue (40.61%), and high change fatigue (19.07%). The unordered multicategory logistic regression showed that hospital grade, monthly income, willingness to learn information technology for “Internet+Nursing Services,” and professional identity in “Internet+Nursing Services” were statistically significant (*p*<0.05).

**Conclusion:**

There is heterogeneity in nurses’ change fatigue within the “Internet+Nursing Services” model. Managers should pay greater attention to nurses experiencing high levels of change fatigue and mitigate this fatigue by strengthening structured empowerment and enhancing their professional identity.

## Introduction

As information technology has advanced rapidly in recent years, healthcare services have shifted to digitalization and platform-based models, resulting in the emergence of the “Internet+Nursing Services” model (1). “Internet+Nursing Services” refers to a new nursing service model in which medical institutions utilize internet technology to integrate online platforms with offline nursing care (2). Although this model provides patients with a more convenient means of accessing care, it also presents nurses with new challenges and pressures. The implementation of new technologies, increased workload, changes in job responsibilities, processes, and role definitions have further exacerbated nurses’ change fatigue (3, 4). A mixed-methods study indicates that healthcare personnel frequently experience change fatigue during digital transformation processes (5). Change fatigue refers to the stress, burnout, and exhaustion resulting from rapid, persistent workplace changes (6). Research indicates that 81.4% of nurses experience change fatigue, approximately three times the rate among other healthcare personnel (7, 8). This fatigue harms nurses’ physical and mental health, reduces their commitment to work, increases job burnout, and affects the quality of care (9, 10). Currently, the number of institutions and nurses participating in China’s “Internet+Nursing Services” has been steadily increasing (11). It suggests that nurses’ change fatigue is likely to become increasingly severe. However, no studies have yet investigated the level of nurses’ change fatigue in “Internet+Nursing Services.”

To mitigate the negative impact of nurses’ change fatigue in “Internet+Nursing Services,” it is necessary to further explore the factors that influence it. The job demand-resource model provides an important theoretical framework for change fatigue. This model includes both health-impairment and incentive paths. When the demands of their jobs exceed available resources, nurses undergo continuous energy depletion, which leads to cumulative stress and exhaustion. High levels of job resources stimulate positive motivation and work engagement, mitigating the negative effects of job demands (12). Job resources refer to physical, psychological, social, and organizational characteristics that provide motivation and support in the workplace (13). Professional identity is a key motivator of nurses’ willingness to participate in “Internet+Nursing Services,” with significant practical implications. Professional identity in “Internet+Nursing Services” serves as a vital job resource, representing an individual’s level of recognition toward their role in this field. It effectively motivates individuals to engage more actively in their work (14). Research indicates that a positive work attitude alleviates change fatigue (15). Therefore, this study hypothesizes that professional identity in “Internet+Nursing Services” can enhance intrinsic motivation and influence change fatigue.

Current research on change fatigue primarily focuses on cross-sectional designs, which neglect group heterogeneity and individual differences. This conclusion limits the scientific assessment and personalized interventions for nurses’ change fatigue among different groups. Additionally, the heterogeneity of nurses’ change fatigue in the “Internet+Nursing Services” model has received insufficient attention. Latent profile analysis is an individual-centered classification method that accurately identifies within-group differences (16). This study uses latent profile analysis to explore the categorical characteristics of change fatigue among nurses in “Internet+Nursing Services,” identify factors influencing these latent categories, and provide evidence for the development of scientifically effective management measures to mitigate change fatigue.

## Materials and methods

### Participants

Using convenience sampling, researchers selected 650 nurses providing “Internet+Nursing Services” from five hospitals in Lishui City as survey subjects between March and April 2025. The selection criteria were as follows: 1) Possess a valid nursing license; 2) With hospital approval, nurses engaged in “Internet+Nursing Services”; 3) Providing an informed consent form and voluntarily participating in this study. The exclusion criteria are as follows: Nurses who were on extended leave, such as sick or maternity leave. The sample size should be at least 5–10 times the number of questionnaire items (17). This study consisted of 49 items. Considering the possibility of invalid entries, the sample size was increased by 20%, with a minimum of 307. Ultimately, 650 nurses participated in the study. The Ethics Committee of Lishui University has approved this study protocol. Approval Number: [2025R006].

## Methods

### General information questionnaire

Researchers designed the study based on prior literature and group discussions. 1) Demographic information: sex, age, level of education, marital status, job title, number of children, position, department, number of nightshifts, years of work experience, hospital grade, monthly income, and specialist nurse. 2) “Internet+Nursing Services” information: travel times, systematic training, family attitude, years of experience in “Internet+Nursing Services,” number of services, willingness to learn information technology for “internet +nursing services,” The willingness to learn information technology for “Internet+Nursing Services” is rated on a five-point scale, where 1 indicates “very unwilling,” and 5 indicates “very willing.”

### Change fatigue scale

The Change Fatigue Scale (CFS) was designed in 2011 by Bernerth et al. (18), based on theories of stress and organizational change. The Chinese version of the CFS was translated by Zhang Xinyue et al. (19) and was used to assess clinical nurses’ change fatigue. The unidimensional scale contains 6 items and is scored on a 7-point Likert scale from “strongly disagree” to “strongly agree.” The higher the total score, the greater the nurses’ change fatigue. The Cronbach’s alpha coefficient for the Chinese version of the CFS is 0.918, the split-half reliability is 0.911, and the test–retest reliability is 0.894. The Cronbach’s alpha coefficient was 0.868 in this study.

### “Internet+Nursing Services” professional identity scale

The “Internet+Nursing Services” professional identity scale, developed by Zhao Liuhong et al. (11), was used to assess nurses’ levels of professional identity in this context. It has been applied among practitioners engaged in “Internet+Nursing Services.” This scale consists of 25 items across four dimensions: professional commitment, professional status, professional cognition, and professional support. It is scored on a 5-point Likert scale from “strongly disagree” to “strongly agree,” with a total score range of 25–125 points. Low score group (25–49 points), relatively low score group (50–74), moderate score group (75–99), and high score group (100–125). The higher the score, the stronger the nurses’ professional identity with “Internet+Nursing Services.” The Cronbach’s alpha coefficient is 0.920 for this scale. The Cronbach’s alpha coefficient was 0.974 in this study.

### Date collection

The questionnaire was created on the Wenjuanxing platform and then distributed by the Lishui City Nursing Association, which provided the Wenjuanxing QR code to nursing work groups. The questionnaire is completed anonymously. Before completion, survey participants are provided with a standardized explanation of the study’s purpose, significance, completion requirements, and commitment to confidentiality, as well as a clear definition of “change fatigue.” To ensure data quality, each mobile phone or computer is allowed to submit the questionnaire only once. This study collected 743 questionnaires. After excluding responses with repetitive patterns, contradictory answers, or completion times under 122 s, 650 valid responses remained, yielding an effective response rate of 87.48%.

### Statistical analysis

Latent profile analysis was used to assess nurses’ change fatigue in “Internet+Nursing Services,” with all analyses performed in Mplus 8.3. In this analysis, a robust maximum likelihood (MLR) method was used to generate a series of LPA models with progressively increasing numbers of latent categories until adding more categories no longer improved the model’s explanatory power. The model fit indices included Akaike’s Information Criterion (AIC), Bayesian Information Criterion (BIC), sample-size-adjusted BIC (aBIC), entropy, the Lo–Mendell–Rubin likelihood ratio test (LMR), and the bootstrap likelihood ratio test (BLRT). Lower AIC, BIC, and aBIC values indicate a superior model fit. The entropy value ranges from 0 to 1. A higher entropy value indicates that classification is more accurate. A statistically significant *p*-value (*p* < 0.05) in the LMR and BLRT tests suggests that the model with k classes fits better than the model with k–1 classes. To select the optimal model, both the interpretability and the practical significance of the latent classes were considered. Statistical analysis was performed using SPSS 27.0. Data following a normal distribution were reported as mean ± standard deviation. Data not following a normal distribution were described using the median [M (P25, P75)]. Intergroup comparisons were performed using the nonparametric rank-sum test. Count data are expressed using case numbers and percentages. Comparisons between groups were performed using chi-square tests. Using variables that showed statistical significance in the univariate analysis as independent variables and latent profiles as dependent variables, an unordered multinomial logistic regression was performed to examine the influence of these factors, with a significance level of *α* = 0.05.

## Results

### General information for nurses in “Internet+Nursing Services”

This study included 650 nurses engaged in “Internet+Nursing Services,” of whom 10 were male (1.5%), and 640 were female (98.5%). Additional details are presented in Table 1.

**Table 1:** General information of survey participants.

Items	Categories	*n*	Percentage (%)	Items	Categories	*n*	Percentage (%)
Age	20–29	90	13.80	Department	Internal medicine	243	37.40
	30–39	338	52		Operating room	132	20.20
	40–49	194	29.80		Emergency department	49	7.50
	≥50	28	4.30		Obstetrics and gynecology	51	7.80
					Pediatrics	36	5.50
Level of education	Junior college	26	4		Others	140	21.50
	Bachelor’s degree or higher	624	96				
				Number of nightshifts (years)	<20	217	41.70
Marital status	Unmarried	90	13.80		20–59	162	24.90
	Married	551	84.80		60–100	168	25.80
	Divorced	9	1.40		>100	49	7.50
Number of children	0	117	18	Years of work experience (years)	<10	114	17.50
	1	271	41.70		10–14	216	33.20
	≥2	262	40.30		15–20	166	25.50
					>20	154	23.70
Position	Nursing management	138	21.20				
	Clinical preceptor	154	23.70	Years of experience in “Internet+Nursing Services.” (years)	<1	77	11.80
	Clinical Nurse	327	50.30		1–2	418	64.30
	Others	31	4.80		>2	155	23.80

### Scores on the change fatigue scale and the “Internet+Nursing Service” professional identity scale

The median total score for nurses’ change fatigue in “Internet+Nursing Services” was 21 (16.00, 26.00). The median total score on professional identity among low-income nurses was 99.50 (86.00, 107.25).

### Potential profile analysis of nurses’ change fatigue in “Internet+Nursing Services”

Using the six items of the change fatigue scale as observed variables, latent class models with 1–4 classes were constructed. Modeling started with a single-class model and progressively increased the number of classes (Table 2). Among the four models, Model 4 had the smallest AIC, BIC, and aBIC values. However, the LMR test was not statistically significant. Model 3 had lower AIC, BIC, and aBIC values, with an entropy of 0.884. In addition, both LMR and BLRT are statistically significant. Model 2 had the highest entropy, and the proportions across all categories were 50%. Therefore, after a comprehensive evaluation, this study selected the 3-class model as the optimal solution. When the number of latent classes was 3, the class probabilities were 97.5, 92.4, and 94.8%, respectively. This indicates high classification accuracy.

**Table 2:** Fitting results for the latent profile model of nurses’ change fatigue in “Internet+Nursing Services.”

Model	AIC	BIC	aBIC	Entropy	LMR	BLRT	Category proportion (%)
1	13,864.469	13,918.193	13,880.093	—	—	—	—
2	12,210.848	12,295.911	12,235.586	0.885	<0.001	<0.001	50/50
3	11,733.978	11,850.379	11,767.83	0.884	0.0006	<0.001	40.30/40.61/19.07
4	11,526.937	11,674.677	11,569.903	0.863	0.2010	<0.001	16.46/37.38/11.53/34.61

### Potential profile characteristics of nurses’ change fatigue in “Internet+Nursing Services”

**Figure 1:**
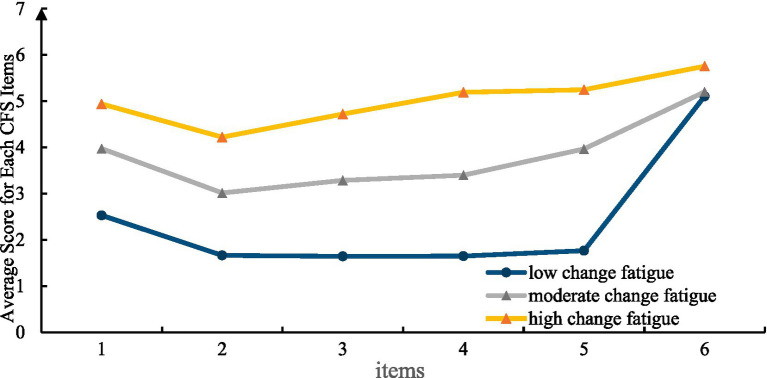
Distribution of potential categories of nurses’ change fatigue in “Internet+Nursing Services.” 1. The organization has introduced too many change measures. 2. I’m tired of all the changes in the organization. 3. The number of changes occurring within the organization is overwhelming. 4. In the organization, we are required to change too many things. 5. It feels like we are constantly being asked to make all kinds of changes. 6. Before implementing any organizational changes, I would like to have a period of stability.

Line charts of CFS item scores were plotted to analyze three latent profiles of nurses’ change fatigue in “Internet+Nursing Services,” as shown in Figure 1. The categories were named based on the fluctuations in the line chart of the mean scores for each item of change fatigue. Category 1, which had significantly lower scores than other categories, was named the “Low Change Fatigue Group” (262 cases, accounting for 40.3%). Category 2, which had mean scores in the moderate range, was named the “Moderate Change Fatigue Group” (264 cases, accounting for 40.61%). The mean scores for all items in Category 3 were significantly higher than those in other categories. Therefore, it was named the “High Change Fatigue Group” (124 cases, accounting for 19.07%).

### Univariate analysis of potential categories of nurses’ change fatigue in “Internet+Nursing Services”

The results indicate that hospital grade, monthly income, systematic training, family attitudes, Willingness to learn information technology for “Internet+Nursing Services,” and professional identity in “Internet+Nursing Services” are statistically significant (*p* < 0.05), as shown in Table 3.

**Table 3:** Univariate analysis of potential categories of nurses’ change fatigue in “Internet +Nursing Services” (*n* = 650).

Classification		Low change fatigue group (*n* = 262)	Moderate change fatigue group (*n* = 264)	High change fatigue group (*n* = 124)	Statistics	*p*
Hospital grade	Level three	168 (64.10)	134 (50.80)	49 (39.50)	26.905	<0.001
	Level two	89 (34)	126 (47.70)	68 (54.80)		
	Community	5 (1.90)	4 (1.50)	7 (5.60)		
Monthly income	<5,000	20 (7.60)	34 (12.90)	28 (22.60)	23.937	<0.001
	5,000–7,999	153 (58.40)	162 (61.40)	74 (59.70)		
	8,000–10,000	72 (27.50)	53 (20.10)	18 (14.50)		
	>10,000	17 (6.50)	15 (5.70)	4 (3.20)		
Specialist nurse	Yes	62 (23.70)	62 (23.50)	22 (17.70)	1.962	0.375
	No	200 (76.30)	202 (76.50)	102 (82.30)		
Job title	Junior professional title	55 (21)	56 (21.20)	27 (21.80)	3.429	0.489
	Intermediate professional title	132 (50.40)	129 (56.40)	69 (55.60)		
	Senior professional title	75 (28.60)	59 (22.30)	28 (22.60)		
Travel times	08:00–12:00	65 (24.80)	50 (18.90)	23 (18.50)	7.989	0.092
	12:00–17:00	78 (29.80)	101 (38.30)	36 (29)		
	After 5:00 p.m.	119 (45.40)	113 (42.80)	65 (52.40)		
Systematic training	Yes	245 (93.50)	233 (88.30)	107 (86.30)	6.376	0.041
	No	17 (6.50)	31 (11.70)	17 (13.70)		
Family attitude	Support	230 (87.80)	172 (65.20)	72 (58.10)	57.030	<0.001
	Neutral	29 (11.10)	87 (33)	44 (35.50)		
	Not supported	3 (1.10)	5 (1.90)	8 (6.50)		
Number of children	0	45 (17.20)	49 (18.60)	23 (18.50)	0.329	0.988
	1	110 (42)	111 (42)	50 (40.30)		
	≥2	107 (40.80)	104 (39.40)	51 (41.10)		
Willing to learn information technology for “Internet+Nursing Services.”		4.29 ± 0.483	3.70 ± 0.516	3.66 ± 0.486	106.742	<0.001
Number of services (points, *x̅* ± s)		10.45 ± 14.625	10.55 ± 22.814	7.31 ± 9.394	2.785	0.248
Professional identity in “Internet+Nursing Services” (score, *x̅* ± s)		110.2 ± 12.943	99.24 ± 13.704	92.07 ± 15.591	133.455	<0.001

### Multifactorial analysis of potential categories of nurses’ change fatigue in “Internet+Nursing Services”

Using the three categories of low, moderate, and high change fatigue as the dependent variable (with the low change fatigue group as the reference), unordered multinomial logistic regression was performed, with statistically significant factors from the univariate analysis as independent variables. SPSS automatically performs categorical data comparisons, so dummy variables are not required. The independent variable values are shown in Table 4. Research indicates that hospital grade, monthly income, willingness to learn about “Internet+Nursing Services,” and professional identity in “Internet+Nursing Services” were factors influencing the latent categories of nurses’ change fatigue in “Internet+Nursing Services,” as shown in Table 5.

**Table 4:** Multivariate logistic regression independent variables assignment.

Variable	Independent variables assignment
Hospital	Level three = 1, level two = 2, community = 3
Monthly income	<5,000 = 1, 5,000–7,999 = 2, 8,000–10,000 = 3, 10,000 = 4
Systematic training	Yes = 1, no = 2
Willing to learn information technology for “Internet+Nursing Services.”	Substitute the original value
Professional identity in “Internet+Nursing Services”	Substitute the original value

**Table 5:** Multifactor analysis of potential categories of nurses’ change fatigue in “Internet+Nursing Services” (*n* = 650).

Items	Moderate change fatigue group				High change fatigue group			
	OR	95% CI	*β*	*p*	OR	95%CI	*β*	*p*
Hospital grade								
Level three	1.167	0.288–4.726	0.155	0.828	0.263	0.070–0.983	−1.337	0.047
Level two	1.884	0.460–7.716	0.634	0.378	0.479	0.127–1.840	−0.735	0.277
Monthly income								
<5,000	1.450	0.514–4.086	0.372	0.482	4.368	1.071–17.808	1.472	0.040
5,000–7,999	1.137	0.494–2.613	0.128	0.763	2.088	0.601–7.248	0.736	0.246
8,000–10,000	0.886	0.366–2.140	−0.122	0.787	1.222	0.324–4.609	0.201	0.767
Systematic training								
Yes	0.905	0.449–1.822	−0.100	0.779	1.153	0.479–2.672	0.142	0.741
Family attitude								
Support	1.528	0.304–7.671	0.424	0.607	0.737	0.139–3.918	−0.305	0.721
Neutral	3.827	0.737–19.859	1.342	0.110	1.712	0.314–9.340	0.538	0.534
Willing to learn information technology for “Internet+Nursing Services.”	0.434	0.313–0.603	−0.834	<0.001	0.624	0.408–0.953	−0.472	0.029
Professional identity in “Internet+Nursing.”	0.972	0.956–0.988	−0.028	<0.001	0.932	0.912–0.953	−0.070	<0.001

1. Hospital grade uses the community as the reference group. 2. Monthly income was categorized, with >10,000 as the reference group. 3. Systematic training uses “no” as the reference group. 4. The family attitude of not supporting is used as a reference group.

## Discussion

### The level of nurses’ change fatigue in the “Internet+Nursing Services” is moderate

The results of this study show that the median total change fatigue score among nurses in “Internet+Nursing Services” was 21 (16.00, 26.00), indicating a moderate level. Similar to the findings of Brown et al. (20), but lower than those of Meng et al. (4) The latter study might be due to the relatively short work experience of most nurses, their limited clinical experience, high learning pressures, and greater susceptibility to change fatigue. In contrast, 82.4% of nurses in this study had worked for more than 10 years. Nurses with extensive experience may be more adaptable, enabling them to respond effectively to organizational change.

### Heterogeneity of nurses’ change fatigue in “Internet+Nursing Services”

Nurses’ change fatigue in “Internet+Nursing Services” was categorized into three groups:

low change fatigue (40.3%), moderate change fatigue (40.61%), and high change fatigue (19.07%). The low change fatigue group is primarily characterized by tertiary hospitals and family support. Tertiary hospitals benefit from policy and technical support, and a collaborative team environment, thereby reducing change fatigue. Additionally, family support provides emotional support, daily care, and role-sharing, alleviating the work–family conflict caused by “Internet+Nursing Services.” Consequently, the level of nurses’ change fatigue remains low. The moderate change fatigue group is primarily characterized by a monthly income of 5,000 to 8,000 yuan. Nurses may have heavy workloads in their departments, and despite their relatively decent income, they are also required to participate in “Internet+Nursing Services.” Therefore, they may experience moderate fatigue. High change fatigue is primarily characterized by hospitals below the tertiary level, lower monthly income, and having two or more children. Nurses in hospitals below the tertiary level are particularly concerned about road safety and medical violence when providing home care, resulting in significant psychological stress (21). They also suffer from insufficient nursing skills; their low monthly income further reduces their motivation to work. Therefore, they experience high levels of work fatigue. Nurses with two or more children spend more time and energy on childcare. Additionally, the occupational health risks and job pressure associated with “Internet+Nursing Services” are relatively high. The combined pressures of job and family life lead to high levels of change fatigue.

The decline from Item 1 to Item 2 across the three categories suggests that although nurses perceive a high degree of organizational change, they do not report feeling significantly fatigued. This means that they may have a relatively high tolerance for change. Item 6, “Before making any changes to the organization, I would like a period of stability”, got a high score from all three groups. This suggests that nurses in the “Internet+Nursing Services” usually desire sufficient time for adaptation and preparation before new changes are implemented. The low change fatigue group showed a greater increase on item 6, consistent with findings from McMillan et al. (22). Nurses experiencing a sense of helplessness and adopting a passive acceptance mindset when confronting rapid and continuous change gradually develop fatigue, burnout, and negative emotions. This effect is powerful when change happens without adequate support or buffering measures, and it can even impact nurses who were initially more positive. Administrators should establish a reasonable transition period based on an assessment of change fatigue. By adjusting the speed of change and adopting a gradual approach, they can help lessen nurses’ exhaustion and resistance.

### Influencing factors of potential categories of nurses’ change fatigue in “Internet+Nursing Services”

#### Hospital grade

Research indicates that compared to the moderate and high change fatigue groups, tertiary hospitals are more likely to fall into the low change fatigue group. This contradicts the findings of Brown et al. (20), who concluded that larger hospital size (number of beds) correlates with increased nursing workload and more severe change fatigue. The reasons for this study may be closely related to disparities in resources among hospitals of different levels within China’s healthcare system. Tertiary hospitals possess distinct advantages in medical resources, technical facilities, and management systems, providing job resources such as information infrastructure, training systems, and organizational support for the change of “Internet+Nursing Services” (23). These resources alleviate stress by enhancing nurses’ work efficiency and professional competencies. They thereby mitigate the negative impact of job demands on psychological exhaustion and occupational burnout, and reduce change fatigue. Past research has shown that change resource support helps alleviate change fatigue (24). Conversely, community hospitals face numerous challenges in practice environments, technical platform development, and specialized training (25). These shortcomings result in nurses lacking the necessary professional skills and resources for “Internet+Nursing Services,” increasing their pressure when facing change. Policy makers should increase financial investment in digital information platforms for primary-level hospitals to narrow resource disparities. Managers should strengthen structural empowerment and establish a systematic training framework that includes digital skills, professional competencies, and emotional intelligence to enhance nurses’ professional expertise and adaptability to change.

#### Monthly income

This study indicates that nurses in the “Internet+Nursing Services” sector with monthly incomes below ¥5,000 are more likely to be classified into the high change fatigue group than those in the low and moderate-change fatigue groups. Which is consistent with the findings of Ming et al. (26). In the context of “Internet+Nursing Services,” nurses face challenges such as patient medical risks, personal safety hazards, and complexities in information communication. Additionally, their time commitment, potential risks, and emotional burden have increased accordingly (27). Other studies found that 90.7% of nurses in “Internet+Nursing Services” did not receive additional income (28). Although nurses participating in the “Internet+Nursing Services” in this study receive some financial compensation, this compensation may be insufficient to offset their additional work input and associated risks. The economic incentive system for “Internet+Nursing Services” remains imperfect. Moreover, empirical research examining its impact on change fatigue is limited. Further analysis indicates that the professional identity of low-income nurses in this study is moderate, resulting in relatively insufficient work resources. The combination of high work demands and relatively insufficient resources makes low-income nurses more prone to burnout and change fatigue when coping with organizational changes related to “Internet+Nursing Services”. Managers are advised to establish a job allowance system that links service quality and workload, and to improve the performance reward mechanism. Peer support and mindfulness training may enhance nurses’ psychological resilience and resources to help alleviate change fatigue.

### Willingness to learn information technology for “Internet+Nursing Services”

This study indicates that, compared with nurses in the moderate-and high-change fatigue groups, those with a higher willingness to learn information technology for “Internet+Nursing Services” are more likely to belong to the low-change fatigue group. The job demands-resources model posits that personal resources represent an individual’s positive self-assessment of their ability to control and influence their environment (29). According to this theoretical framework, nurses who are willing to learn information technology for “Internet+Nursing Services” are more tend to proactively engage in learning. By continuously accumulating knowledge and skills, they enhance their sense of competence, thereby increasing self-efficacy. A higher sense of self-efficacy leads to greater engagement at work, thereby helping nurses address problems more proactively (30). Against the backdrop of rapid technological development, these nurses can actively learn and master new technologies and skillfully use various digital tools, thereby improving their adaptability to the information-based workload of “Internet+Nursing Services,” reducing anxiety and a sense of powerlessness, and alleviating change fatigue. It is recommended that managers prioritize enhancing their self-efficacy levels, encourage nurses to engage in proactive learning and actively participate in technological innovation, thereby elevating the digital literacy of the nursing workforce and driving the high-quality development of smart nursing.

### Professional identity in “Internet+Nursing Services”

The study indicates that compared to the moderate and high change fatigue groups, nurses with higher levels of professional identity toward “Internet+Nursing Services” are more likely to belong to the low change fatigue group. This finding is consistent with Tarsuslu’s research (31). According to the job demands–resources model, an individual’s perception of job demands and the availability of mobilizable resources jointly influence their coping strategies and behavioral responses (32). Within the context of “internet+ nursing services,” nurses must not only fulfill routine clinical duties but also handle online platform operations and in-home service tasks, which encroach on their personal rest time and further increase their workload and psychological stress. However, when patients and their families acknowledge nurses’ professional abilities, it often strengthens nurses’ sense of self-worth and achievement (33). When nurses feel their work is valued and recognized, it inspires a stronger sense of professional purpose and belonging, which boosts their intrinsic motivation. This results in favorable feedback regarding their work, buffering the physical and mental toll of organizational change, reducing burnout, and alleviating change fatigue. Conversely, when professional identity in “Internet+Nursing Services” is lower, their resilience to work-related stress diminishes, thereby creating a negative cycle of insufficient resources-increased pressure-restricted behavior, thereby heightening the risk of change fatigue. It is recommended that administrators prioritize developing a systematic and diversified incentive system. This system should demonstrate work achievements and value through patient satisfaction metrics, platform honor badges, badge redemption programs, and the visualization of nursing quality data (34). These measures will enhance professional recognition of the “Internet+Nursing Services,” improve the quality of nursing care, and promote the modernization of hospital management.

### Limitations

This study was limited by geographical constraints and only surveyed nurses providing “Internet+Nursing Services” in Lishui City. Future research could be conducted as a multicenter study across multiple provinces to enhance the generalizability of the findings.

Furthermore, this study is a self-reported cross-sectional study, which cannot establish causal relationships and cannot reflect the dynamic changes in nurses’ transformational fatigue. Therefore, we should conduct longitudinal studies to explore the changing trajectory of factors influencing nurses’ change fatigue under the “Internet+Nursing Services” model. We should also combine this with qualitative research to explore the psychological experiences of nurses experiencing change fatigue. Since the questionnaire was distributed through nursing associations, nurses may underestimate their own levels of change fatigue, thereby introducing a social desirability bias. Although this study employed anonymous surveys and incorporated objective workload metrics to mitigate this bias, the findings should be interpreted with caution. Future research may consider distributing questionnaires through non-work-related channels.

## Conclusion

Nurses in “Internet+Nursing Services” experience a moderate level of change fatigue. There are three groups: low change fatigue, moderate change fatigue, and high change fatigue. The proportion of nurses in the high-change fatigue group is lowest, with significant room for improvement. Hospital administrators should focus on individuals in the moderate and high change fatigue groups to target intervention measures for them.

## Data Availability

The raw data supporting the conclusions of this article will be made available by the authors, without undue reservation.
